# 4-(4-Carb­oxy-1,3-thia­zol-2-yl)pyridinium 3-carb­oxy-4-hydroxy­benzene­sulfonate dihydrate

**DOI:** 10.1107/S1600536808030924

**Published:** 2008-09-30

**Authors:** Zhong-Xiang Du, Jun-Xia Li

**Affiliations:** a Department of Chemistry and Chemical Engineering, Luoyang Normal University, Luoyang, Henan 471022, People’s Republic of China

## Abstract

In the crystal structure of the title compound, C_9_H_7_N_2_O_2_S^+^·C_7_H_5_O_6_S^−^·2H_2_O, an H atom from the 5-sulfosalicylic acid is transferred to the pyridyl N atom, forming a salt. The dihedral angle between the thiazole and pyridinium rings is 5.909 (5)°. The crystal packing is determined by O—H⋯O and N—H⋯O hydrogen bonds involving water mol­ecules.

## Related literature

For related structures, see: Chen *et al.* (2007 ▶); Ellsworth *et al.* (2006 ▶); Su *et al.* (2004 ▶).
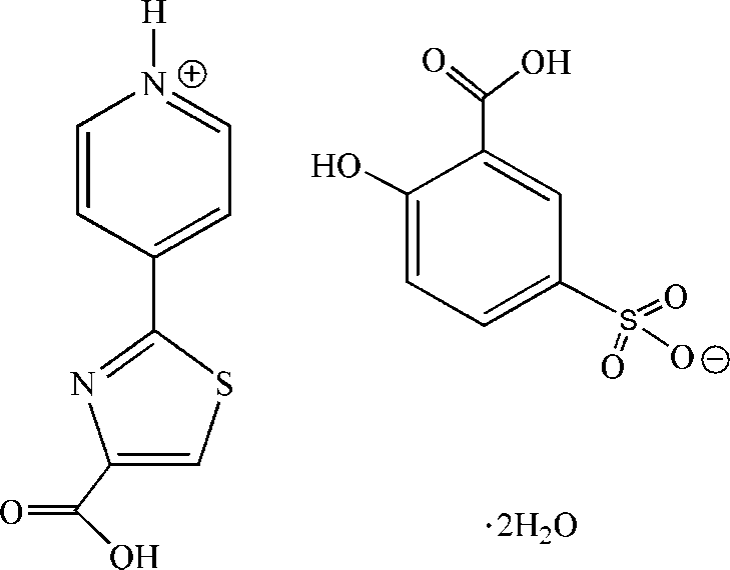


## Experimental

### 

#### Crystal data

C_9_H_7_N_2_O_2_S^+^·C_7_H_5_O_6_S^−^·2H_2_O
*M* *_r_* = 460.43
Triclinic, 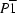

*a* = 8.6234 (14) Å
*b* = 10.6065 (17) Å
*c* = 10.7979 (17) Å
α = 97.799 (2)°
β = 94.479 (2)°
γ = 99.885 (2)°
*V* = 958.7 (3) Å^3^
*Z* = 2
Mo *K*α radiation
μ = 0.34 mm^−1^
*T* = 291 (2) K
0.44 × 0.29 × 0.24 mm

#### Data collection

Bruker APEXII CCD area-detector diffractometer
Absorption correction: multi-scan (*SADABS*; Sheldrick, 1996 ▶) *T*_min_ = 0.867, *T*_max_ = 0.924
7016 measured reflections
3494 independent reflections
3095 reflections with *I* > 2σ(*I*)
*R*_int_ = 0.014

#### Refinement

*R*[*F* ^2^ > 2σ(*F* ^2^)] = 0.032
*wR*(*F* ^2^) = 0.092
*S* = 1.03
3494 reflections
275 parameters
6 restraints
H-atom parameters constrained
Δρ_max_ = 0.31 e Å^−3^
Δρ_min_ = −0.29 e Å^−3^

### 

Data collection: *APEX2* (Bruker, 2004 ▶); cell refinement: *APEX2*; data reduction: *SAINT* (Bruker, 2004 ▶); program(s) used to solve structure: *SHELXS97* (Sheldrick, 2008 ▶); program(s) used to refine structure: *SHELXL97* (Sheldrick, 2008 ▶); molecular graphics: *SHELXTL* (Sheldrick, 2008 ▶); software used to prepare material for publication: *SHELXTL*.

## Supplementary Material

Crystal structure: contains datablocks global, I. DOI: 10.1107/S1600536808030924/kp2193sup1.cif

Structure factors: contains datablocks I. DOI: 10.1107/S1600536808030924/kp2193Isup2.hkl

Additional supplementary materials:  crystallographic information; 3D view; checkCIF report

## Figures and Tables

**Table 1:** Hydrogen-bond geometry (Å, °)

*D*—H⋯*A*	*D*—H	H⋯*A*	*D*⋯*A*	*D*—H⋯*A*
O1—H1⋯O2	0.82	1.88	2.599 (2)	146
O3—H3⋯O9	0.82	1.71	2.5269 (17)	171
O8—H8⋯O4^i^	0.82	1.89	2.6979 (18)	171
O9—H1*W*⋯O6^ii^	0.84	1.93	2.753 (2)	165
O9—H2*W*⋯O5^iii^	0.83	1.89	2.713 (2)	172
O10—H3*W*⋯O2	0.81	2.32	2.9001 (19)	129
O10—H4*W*⋯O7^iv^	0.81	2.27	2.835 (2)	128
N2—H2*D*⋯O10^v^	0.86	1.86	2.689 (2)	162

Symmetry codes: (i) 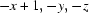; (ii) 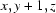; (iii) 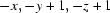; (iv) 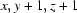; (v) 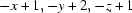.

**Fig. 1.**
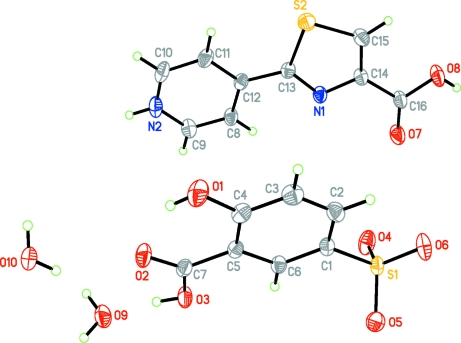
Molecular structure of (I), with displacement ellipsoids drawn at the 30% probability level.

**Fig. 2.**
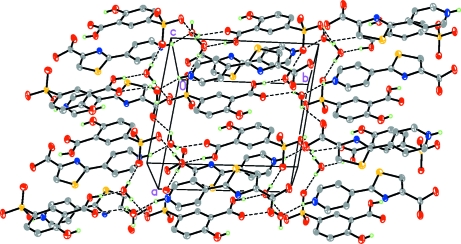
The crystal packing of (I), showing hydrogen bonds as dashed lines. For the sake of clarity, H atoms on C atoms have been omitted.

## supplementary crystallographic information

### Comment

2-(4-Pyridyl)thiazole-4-carboxylic acid (HPTCA), which is an asymmetric, chelating ligand, has been studied in recent years. Five of its transition metal complexes (Chen *et al.*, 2007; Ellsworth *et al.*, 2006; Su *et al.*, 2004) were reported. In this paper we describe its salt with 5-sulfosalicylic acid (H_3_SSA), (I).

The crystal structure of the title molecule comprises 2-(4-pyridylomium)thiazole-4-carboxylic acid, a 5-sulfosalicylic acid anion and two water molecules (Fig.1). The H atom of the 5-sulfosalicylic acid is transferred to the pyridyl N-atom of 2-(4-pyridyl)thiazole-4-carboxylic acid, thus forming a salt. The dihedral angle between the thiazole and pyridinium rings is 5.909 (5)°. The N—H and O—H groups  are involved in intra- and intermolecular hydrogen bonds with water molecules generating the 3-dimensional hydrogen bond network (Table 1 and Fig. 2).

### Experimental

The ligand HPTCA (1 mmol, 0.21 g) and H_3_SSA.2H_2_O (1 mmol, 0.25 g) were dissolved in solvent mixture of water and methanol (20 mL, *v*/*v* 1:1). To this solution, Cu(CH_3_COO)_2_.4H_2_O (1 mmol, 0.26 g) was added and the resulting mixture was stirred and refluxed at 353 K for 3 h, then cooled to room temperature. After filtration and evaporation in air for five days, colourless claviform-shaped crystals were obtained in a yield of 43%. Analysis, found (%): C, 41.75; H, 3.51; N, 6.02; S,13.87. C_16_H_16_N_2_O_10_S_2_ requires (%): C,41.70; H,3.47; N,6.08; S,13.90. (The elemental analysis indicates that the copper(II) is not coordinated by the ligands) (CCDC number 685021)

### Refinement

H Atoms bonded to C or N atoms were positioned geometrically with C—H distance of 0.93Å and N—H distance of 0.86 Å, and treated as riding atoms, with *U*_iso_(H)=1.2*U*_eq_(C or N). H atoms bonded to O atoms were located in a difference Fourier map and refined isotropically.

### Crystal data

**Table:**

C_9_H_7_N_2_O_2_S^+^·C_7_H_5_O_6_S^−^·2H_2_O	*Z* = 2
*M**_r_* = 460.43	*F*(000) = 476
Triclinic, *P*1	*D*_x_ = 1.595 Mg m^−^^3^
Hall symbol: -P 1	Mo *K*α radiation, λ = 0.71073 Å
*a* = 8.6234 (14) Å	Cell parameters from 4068 reflections
*b* = 10.6065 (17) Å	θ = 2.4–28.1°
*c* = 10.7979 (17) Å	µ = 0.34 mm^−^^1^
α = 97.799 (2)°	*T* = 291 K
β = 94.479 (2)°	Claviform, colourless
γ = 99.885 (2)°	0.44 × 0.29 × 0.24 mm
*V* = 958.7 (3) Å^3^	

### Data collection

**Table:**

Bruker APEXII CCD area-detector diffractometer	3494 independent reflections
Radiation source: fine-focus sealed tube	3095 reflections with *I* > 2σ(*I*)
graphite	*R*_int_ = 0.014
φ and ω scans	θ_max_ = 25.5°, θ_min_ = 2.4°
Absorption correction: multi-scan (SADABS; Sheldrick, 1996)	*h* = −10→10
*T*_min_ = 0.867, *T*_max_ = 0.924	*k* = −12→12
7016 measured reflections	*l* = −13→13

### Refinement

**Table:**

Refinement on *F*^2^	Secondary atom site location: difference Fourier map
Least-squares matrix: full	Hydrogen site location: inferred from neighbouring sites
*R*[*F*^2^ > 2σ(*F*^2^)] = 0.032	H-atom parameters constrained
*wR*(*F*^2^) = 0.092	*w* = 1/[σ^2^(*F*_o_^2^) + (0.0504*P*)^2^ + 0.314*P*] where *P* = (*F*_o_^2^ + 2*F*_c_^2^)/3
*S* = 1.03	(Δ/σ)_max_ < 0.001
3494 reflections	Δρ_max_ = 0.31 e Å^−^^3^
275 parameters	Δρ_min_ = −0.29 e Å^−^^3^
6 restraints	Extinction correction: SHELXL97, Fc^*^=kFc[1+0.001xFc^2^λ^3^/sin(2θ)]^-1/4^
Primary atom site location: structure-invariant direct methods	Extinction coefficient: 0.021 (2)

### Special details

**Table:**

Geometry. All e.s.d.'s (except the e.s.d. in the dihedral angle between two l.s. planes)are estimated using the full covariance matrix. The cell e.s.d.'s are takeninto account individually in the estimation of e.s.d.'s in distances, anglesand torsion angles; correlations between e.s.d.'s in cell parameters are onlyused when they are defined by crystal symmetry. An approximate (isotropic)treatment of cell e.s.d.'s is used for estimating e.s.d.'s involving l.s. planes.
Refinement. Refinement of *F*^2^ against ALL reflections. The weighted *R*-factor *wR* andgoodness of fit *S* are based on *F*^2^, conventional *R*-factors *R* are basedon *F*, with *F* set to zero for negative *F*^2^. The threshold expression of*F*^2^ > σ(*F*^2^) is used only for calculating *R*-factors(gt) *etc*. and isnot relevant to the choice of reflections for refinement. *R*-factors basedon *F*^2^ are statistically about twice as large as those based on *F*, and *R*-factors based on ALL data will be even larger.

### Fractional atomic coordinates and isotropic or equivalent isotropic displacement parameters (Å^2^)

**Table:**

	*x*	*y*	*z*	*U*_iso_*/*U*_eq_	
S1	0.25056 (5)	0.11285 (4)	0.35763 (4)	0.03667 (14)	
S2	1.01768 (5)	0.41512 (5)	0.19381 (4)	0.04482 (15)	
O1	0.52983 (18)	0.53943 (14)	0.76634 (12)	0.0532 (4)	
H1	0.4963	0.6058	0.7574	0.080*	
O2	0.34176 (16)	0.67695 (12)	0.67191 (12)	0.0472 (3)	
O3	0.18259 (15)	0.59188 (11)	0.49653 (12)	0.0432 (3)	
H3	0.1683	0.6667	0.4998	0.065*	
O4	0.30140 (18)	0.15439 (12)	0.24126 (12)	0.0513 (4)	
O5	0.07938 (16)	0.09571 (14)	0.35499 (15)	0.0595 (4)	
O6	0.31219 (19)	0.00026 (13)	0.38592 (14)	0.0577 (4)	
O7	0.61923 (18)	0.12933 (14)	−0.10879 (16)	0.0643 (4)	
O8	0.81979 (18)	0.03591 (13)	−0.04892 (13)	0.0525 (4)	
H8	0.7748	−0.0241	−0.1027	0.079*	
O9	0.11825 (18)	0.81397 (13)	0.48548 (15)	0.0566 (4)	
H1W	0.1851	0.8757	0.4688	0.085*	
H2W	0.0648	0.8431	0.5397	0.085*	
O10	0.35251 (19)	0.94663 (14)	0.77184 (18)	0.0746 (5)	
H3W	0.3066	0.8901	0.7160	0.112*	
H4W	0.4477	0.9618	0.7742	0.112*	
N1	0.77420 (17)	0.36608 (13)	0.03035 (13)	0.0356 (3)	
N2	0.7588 (2)	0.83610 (15)	0.15551 (16)	0.0488 (4)	
H2D	0.7349	0.9120	0.1660	0.059*	
C1	0.33096 (19)	0.24035 (15)	0.48162 (15)	0.0324 (4)	
C2	0.4457 (2)	0.22332 (18)	0.57360 (17)	0.0398 (4)	
H2	0.4793	0.1444	0.5709	0.048*	
C3	0.5089 (2)	0.32448 (19)	0.66860 (17)	0.0428 (4)	
H3A	0.5849	0.3130	0.7300	0.051*	
C4	0.4600 (2)	0.44306 (17)	0.67308 (15)	0.0368 (4)	
C5	0.34238 (19)	0.46005 (15)	0.58262 (14)	0.0310 (3)	
C6	0.27899 (19)	0.35698 (15)	0.48679 (15)	0.0312 (3)	
H6	0.2013	0.3672	0.4261	0.037*	
C7	0.28871 (19)	0.58539 (16)	0.58768 (15)	0.0336 (4)	
C8	0.7104 (2)	0.62363 (17)	0.04650 (16)	0.0396 (4)	
H8A	0.6538	0.5612	−0.0173	0.048*	
C9	0.6759 (2)	0.74537 (19)	0.06439 (18)	0.0473 (5)	
H9	0.5946	0.7655	0.0132	0.057*	
C10	0.8772 (3)	0.81171 (18)	0.23019 (19)	0.0517 (5)	
H10	0.9334	0.8769	0.2917	0.062*	
C11	0.9166 (2)	0.69063 (18)	0.21681 (18)	0.0456 (4)	
H11	0.9993	0.6738	0.2688	0.055*	
C12	0.8314 (2)	0.59344 (16)	0.12459 (15)	0.0342 (4)	
C13	0.8626 (2)	0.46044 (16)	0.10910 (15)	0.0336 (4)	
C14	0.8288 (2)	0.25269 (16)	0.03559 (15)	0.0354 (4)	
C15	0.9591 (2)	0.26100 (18)	0.11859 (17)	0.0420 (4)	
H15	1.0088	0.1921	0.1320	0.050*	
C16	0.7430 (2)	0.13402 (17)	−0.04781 (17)	0.0402 (4)	

### Atomic displacement parameters (Å^2^)

**Table:**

	*U*^11^	*U*^22^	*U*^33^	*U*^12^	*U*^13^	*U*^23^
S1	0.0437 (3)	0.0231 (2)	0.0418 (3)	0.00772 (17)	0.00411 (18)	−0.00162 (17)
S2	0.0427 (3)	0.0475 (3)	0.0405 (3)	0.0097 (2)	−0.01003 (19)	0.0000 (2)
O1	0.0644 (9)	0.0472 (8)	0.0389 (7)	0.0037 (7)	−0.0181 (6)	−0.0035 (6)
O2	0.0590 (8)	0.0326 (7)	0.0432 (7)	0.0049 (6)	−0.0022 (6)	−0.0095 (6)
O3	0.0501 (7)	0.0283 (6)	0.0480 (7)	0.0111 (5)	−0.0082 (6)	−0.0028 (5)
O4	0.0753 (10)	0.0361 (7)	0.0368 (7)	0.0011 (6)	0.0047 (6)	−0.0024 (5)
O5	0.0440 (8)	0.0457 (8)	0.0774 (10)	0.0017 (6)	0.0034 (7)	−0.0207 (7)
O6	0.0805 (10)	0.0317 (7)	0.0647 (9)	0.0235 (7)	0.0058 (8)	0.0046 (6)
O7	0.0581 (9)	0.0427 (8)	0.0821 (11)	0.0191 (7)	−0.0240 (8)	−0.0207 (7)
O8	0.0696 (9)	0.0377 (7)	0.0499 (8)	0.0260 (7)	−0.0084 (7)	−0.0067 (6)
O9	0.0660 (9)	0.0342 (7)	0.0736 (10)	0.0169 (6)	0.0145 (7)	0.0081 (7)
O10	0.0556 (9)	0.0383 (8)	0.1174 (14)	0.0034 (7)	0.0108 (9)	−0.0263 (8)
N1	0.0414 (8)	0.0307 (7)	0.0332 (7)	0.0090 (6)	−0.0031 (6)	0.0001 (6)
N2	0.0659 (11)	0.0289 (8)	0.0519 (10)	0.0092 (7)	0.0134 (8)	0.0022 (7)
C1	0.0370 (9)	0.0281 (8)	0.0322 (8)	0.0062 (7)	0.0051 (7)	0.0034 (7)
C2	0.0419 (10)	0.0378 (9)	0.0430 (10)	0.0134 (8)	0.0039 (8)	0.0106 (8)
C3	0.0404 (10)	0.0505 (11)	0.0380 (9)	0.0094 (8)	−0.0043 (7)	0.0126 (8)
C4	0.0390 (9)	0.0404 (9)	0.0278 (8)	0.0010 (7)	0.0002 (7)	0.0039 (7)
C5	0.0338 (8)	0.0298 (8)	0.0281 (8)	0.0026 (7)	0.0052 (6)	0.0028 (6)
C6	0.0342 (8)	0.0295 (8)	0.0285 (8)	0.0053 (6)	−0.0008 (6)	0.0027 (6)
C7	0.0359 (8)	0.0297 (8)	0.0321 (8)	0.0002 (7)	0.0055 (7)	0.0003 (7)
C8	0.0465 (10)	0.0344 (9)	0.0352 (9)	0.0076 (8)	−0.0003 (7)	−0.0023 (7)
C9	0.0567 (12)	0.0403 (10)	0.0457 (11)	0.0141 (9)	0.0041 (9)	0.0033 (8)
C10	0.0633 (13)	0.0330 (10)	0.0494 (11)	−0.0050 (9)	0.0027 (10)	−0.0075 (8)
C11	0.0500 (11)	0.0388 (10)	0.0412 (10)	0.0005 (8)	−0.0061 (8)	−0.0026 (8)
C12	0.0398 (9)	0.0319 (9)	0.0286 (8)	0.0022 (7)	0.0049 (7)	0.0009 (7)
C13	0.0374 (9)	0.0341 (9)	0.0277 (8)	0.0054 (7)	0.0006 (7)	0.0024 (7)
C14	0.0416 (9)	0.0334 (9)	0.0325 (8)	0.0118 (7)	0.0026 (7)	0.0036 (7)
C15	0.0448 (10)	0.0421 (10)	0.0411 (10)	0.0171 (8)	0.0002 (8)	0.0043 (8)
C16	0.0475 (10)	0.0329 (9)	0.0407 (10)	0.0147 (8)	0.0007 (8)	−0.0001 (7)

### Geometric parameters (Å, °)

**Table:**

S1—O6	1.4469 (14)	C1—C6	1.382 (2)
S1—O5	1.4535 (14)	C1—C2	1.398 (2)
S1—O4	1.4613 (14)	C2—C3	1.383 (3)
S1—C1	1.7731 (17)	C2—H2	0.9300
S2—C15	1.6968 (19)	C3—C4	1.390 (3)
S2—C13	1.7334 (17)	C3—H3A	0.9300
O1—C4	1.355 (2)	C4—C5	1.404 (2)
O1—H1	0.8200	C5—C6	1.401 (2)
O2—C7	1.234 (2)	C5—C7	1.476 (2)
O3—C7	1.308 (2)	C6—H6	0.9300
O3—H3	0.8200	C8—C9	1.366 (3)
O7—C16	1.199 (2)	C8—C12	1.397 (2)
O8—C16	1.325 (2)	C8—H8A	0.9300
O8—H8	0.8200	C9—H9	0.9300
O9—H1W	0.8436	C10—C11	1.376 (3)
O9—H2W	0.8319	C10—H10	0.9300
O10—H3W	0.8145	C11—C12	1.393 (2)
O10—H4W	0.8065	C11—H11	0.9300
N1—C13	1.308 (2)	C12—C13	1.471 (2)
N1—C14	1.371 (2)	C14—C15	1.365 (2)
N2—C10	1.333 (3)	C14—C16	1.487 (2)
N2—C9	1.342 (3)	C15—H15	0.9300
N2—H2D	0.8600		
			
O6—S1—O5	113.03 (9)	C1—C6—H6	119.7
O6—S1—O4	112.45 (9)	C5—C6—H6	119.7
O5—S1—O4	110.37 (9)	O2—C7—O3	122.98 (16)
O6—S1—C1	106.53 (8)	O2—C7—C5	121.86 (16)
O5—S1—C1	106.90 (8)	O3—C7—C5	115.17 (14)
O4—S1—C1	107.15 (8)	C9—C8—C12	119.66 (17)
C15—S2—C13	89.46 (8)	C9—C8—H8A	120.2
C4—O1—H1	109.5	C12—C8—H8A	120.2
C7—O3—H3	109.5	N2—C9—C8	120.27 (18)
C16—O8—H8	109.5	N2—C9—H9	119.9
H1W—O9—H2W	108.7	C8—C9—H9	119.9
H3W—O10—H4W	115.9	N2—C10—C11	120.40 (17)
C13—N1—C14	110.35 (14)	N2—C10—H10	119.8
C10—N2—C9	121.80 (17)	C11—C10—H10	119.8
C10—N2—H2D	119.1	C10—C11—C12	119.39 (18)
C9—N2—H2D	119.1	C10—C11—H11	120.3
C6—C1—C2	120.15 (15)	C12—C11—H11	120.3
C6—C1—S1	119.39 (12)	C11—C12—C8	118.45 (16)
C2—C1—S1	120.46 (13)	C11—C12—C13	122.13 (16)
C3—C2—C1	119.70 (16)	C8—C12—C13	119.41 (15)
C3—C2—H2	120.2	N1—C13—C12	122.45 (15)
C1—C2—H2	120.2	N1—C13—S2	114.42 (13)
C2—C3—C4	120.60 (16)	C12—C13—S2	123.13 (12)
C2—C3—H3A	119.7	N1—C14—C15	115.53 (16)
C4—C3—H3A	119.7	N1—C14—C16	118.19 (14)
O1—C4—C3	117.74 (15)	C15—C14—C16	126.27 (16)
O1—C4—C5	122.23 (16)	C14—C15—S2	110.25 (13)
C3—C4—C5	120.04 (16)	C14—C15—H15	124.9
C4—C5—C6	118.92 (15)	S2—C15—H15	124.9
C4—C5—C7	120.28 (15)	O7—C16—O8	124.15 (17)
C6—C5—C7	120.79 (15)	O7—C16—C14	123.19 (16)
C1—C6—C5	120.57 (15)	O8—C16—C14	112.65 (15)
			
O6—S1—C1—C6	−173.04 (13)	C12—C8—C9—N2	0.8 (3)
O5—S1—C1—C6	−51.93 (16)	C9—N2—C10—C11	−0.9 (3)
O4—S1—C1—C6	66.39 (15)	N2—C10—C11—C12	−0.1 (3)
O6—S1—C1—C2	6.85 (17)	C10—C11—C12—C8	1.5 (3)
O5—S1—C1—C2	127.96 (15)	C10—C11—C12—C13	−177.15 (17)
O4—S1—C1—C2	−113.72 (15)	C9—C8—C12—C11	−1.8 (3)
C6—C1—C2—C3	−1.1 (3)	C9—C8—C12—C13	176.87 (16)
S1—C1—C2—C3	179.01 (13)	C14—N1—C13—C12	−179.49 (15)
C1—C2—C3—C4	−0.3 (3)	C14—N1—C13—S2	0.04 (18)
C2—C3—C4—O1	−178.07 (16)	C11—C12—C13—N1	173.78 (16)
C2—C3—C4—C5	1.7 (3)	C8—C12—C13—N1	−4.8 (2)
O1—C4—C5—C6	178.11 (15)	C11—C12—C13—S2	−5.7 (2)
C3—C4—C5—C6	−1.6 (2)	C8—C12—C13—S2	175.67 (13)
O1—C4—C5—C7	−0.9 (3)	C15—S2—C13—N1	−0.04 (14)
C3—C4—C5—C7	179.36 (15)	C15—S2—C13—C12	179.49 (15)
C2—C1—C6—C5	1.1 (2)	C13—N1—C14—C15	0.0 (2)
S1—C1—C6—C5	−178.97 (12)	C13—N1—C14—C16	−179.94 (15)
C4—C5—C6—C1	0.2 (2)	N1—C14—C15—S2	0.0 (2)
C7—C5—C6—C1	179.24 (14)	C16—C14—C15—S2	179.90 (15)
C4—C5—C7—O2	−1.7 (2)	C13—S2—C15—C14	0.02 (14)
C6—C5—C7—O2	179.26 (15)	N1—C14—C16—O7	−8.5 (3)
C4—C5—C7—O3	177.84 (15)	C15—C14—C16—O7	171.6 (2)
C6—C5—C7—O3	−1.2 (2)	N1—C14—C16—O8	170.21 (15)
C10—N2—C9—C8	0.6 (3)	C15—C14—C16—O8	−9.7 (3)

### Hydrogen-bond geometry (Å, °)

**Table:**

*D*—H···*A*	*D*—H	H···*A*	*D*···*A*	*D*—H···*A*
O1—H1···O2	0.82	1.88	2.599 (2)	146.
O3—H3···O9	0.82	1.71	2.5269 (17)	171.
O8—H8···O4^i^	0.82	1.89	2.6979 (18)	171.
O9—H1W···O6^ii^	0.84	1.93	2.753 (2)	165.
O9—H2W···O5^iii^	0.83	1.89	2.713 (2)	172.
O10—H3W···O2	0.81	2.32	2.9001 (19)	129.
O10—H4W···O7^iv^	0.81	2.27	2.835 (2)	128.
N2—H2D···O10^v^	0.86	1.86	2.689 (2)	162.

Symmetry codes: (i) −*x*+1, −*y*, −*z*; (ii) *x*, *y*+1, *z*; (iii) −*x*, −*y*+1, −*z*+1; (iv) *x*, *y*+1, *z*+1; (v) −*x*+1, −*y*+2, −*z*+1.
